# User Experience of and Adherence to a Smartphone App to Maintain Behavior Change and Self-Management in Patients With Work-Related Skin Diseases: Multistep, Single-Arm Feasibility Study

**DOI:** 10.2196/66791

**Published:** 2025-04-18

**Authors:** Nele Ristow, Annika Wilke, Christoph Skudlik, Swen Malte John, Michaela Ludewig

**Affiliations:** 1 Department of Dermatology, Environmental Medicine and Health Theory Institute for Health Research and Education Osnabrück University Osnabrück Germany; 2 Institute for Interdisciplinary Dermatological Prevention and Rehabilitation (iDerm) at the Osnabrück University Osnabrück Germany; 3 Department Health Sciences Hochschule Bochum Bochum Germany

**Keywords:** user experience, mobile health, mHealth, app, smartphone, complex intervention, Template for Intervention Description and Replication, behavior change techniques, behavior change, skin diseases, occupational dermatology, artificial intelligence

## Abstract

**Background:**

Smartphone apps are a growing field supporting the prevention of chronic diseases. The user experience (UX) is an important predictor of app use and should be considered in mobile health research. Long-term skin protection behavior is important for those with work-related skin diseases. However, altering health behavior is complex and requires a high level of self-management. We developed a maintenance program consisting of the Mein Hautschutz im Alltag (MiA; “My skin protection in everyday life”) app combined with an individual face-to-face goal-setting interview to support patients in the implementation of skin protection behavior after inpatient rehabilitation.

**Objective:**

The objectives of this paper are to (1) describe the intervention in a standardized manner; (2) evaluate the UX, subjective quality, and perceived impact of the MiA app; and (3) evaluate the adherence to the MiA app.

**Methods:**

We followed a user-centered and multistage iterative process in 2 steps that combined qualitative and quantitative data. The maintenance program was tested over 12 weeks after discharge from rehabilitation. The UX, subjective quality, and perceived impact were evaluated formatively based on the user version of the Mobile Application Rating Scale after 12 weeks (T2). Adherence was measured using the frequency of interactions with the app.

**Results:**

In total, 42 patients took part (with a dropout rate of n=18, 43% at T2). The average age was 49.5 (SD 13.1) years, and 57% (24/42) were male. We found high ratings for the UX, with an average score of 80.18 (SD 8.94) out of a theoretical maximum of 100, but there were a few exceptions in the usability and interaction with the app. The app was most frequently rated with 4 out of 5 stars (15/24, 65%), which indicates a high subjective quality. Furthermore, the app seemed to influence important determinants to implement skin protection behavior. Adherence to skin protection tracking was higher over the study period than adherence to skin documentation and goal assessment. The number of adherent participants to skin protection tracking was higher in the skin care and skin cleansing categories (28/42, 67% each) compared to the skin protection category (13/42, 31%) on day 1 and decreased until day 84 in all dimensions (12/42, 29% each for skin care and skin cleansing; 9/42, 21% for skin protection).

**Conclusions:**

The results in terms of adherence met the expectations and were consistent with those of other studies evaluating the use of apps for chronic diseases. Interaction with the app could be increased using artificial intelligence to determine eczema severity via photos. It should be investigated which subgroups have difficulties with usability to individualize the support to a greater degree during onboarding. There is a need for further research regarding the effectiveness of the MiA app on skin protection behavior, quality of life, and eczema severity.

## Introduction

### Background

Smartphone apps have recently become a growing field in the health care sector, garnering increasing attention and importance in health research. The use of such technologies can help optimize broad and location-independent health care in real time and support the prevention of chronic diseases and health promotion to improve adherence to therapy, quality of life, and clinical outcomes in the long term [1,2]. Smartphone apps not only help monitor health data [3,4] but also help change health behavior and develop new habits. For this purpose, health behavior apps offer the potential to technically integrate a variety of behavior change techniques (BCTs) [1,5,6]. BCTs are based on health psychology knowledge about the mechanisms of behavior change processes [7]. The most common BCTs in behavior change apps are, for example, self-monitoring by tracking the duration and frequency of a behavior and feedback on the behavior or outcomes by visualizing the progress graphically [1,5,6,8]. Feedback and the visualization of success also increase motivation in the users, which plays an important role in the long-term success of behavior change [5]. Behavior change apps can also be used to set goals and monitor their achievement. Automatic reminders provide prompts and support the development of routines in everyday life. Written, visual, and audio information can show users how to perform the behavior [1,5,6,8].

### Relevance of User Experience

**Figure 1:**
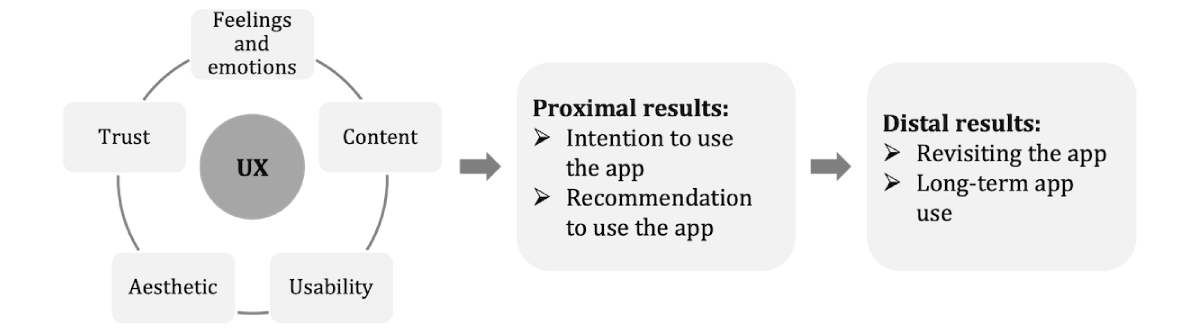
Theoretical model to explain the influence of user experience (UX) and its 5 dimensions—content, usability, aesthetic, trust, and feelings and emotions—on the intention and recommendation to use the app (proximal) results, as well as the revisiting the app and long-term use of the app (distal) results as the basis for this study.

A known and widely discussed problem in research on behavior change apps is a low adherence and engagement when using these technologies [2,9-11]. To address these problems, user experience (UX) is becoming increasingly important in mobile health research [12-15]. UX is an overarching holistic concept and covers all emotional, cognitive, and physical experiences that users undergo when interacting with software products such as apps [12,16-18]. As shown in Figure 1, a high UX when using an app for the first time is associated with the willingness to continue using it and recommend it to others in the short term. A high UX leads to increased adherence and app use in the long term and, thus, can also improve clinical outcomes and the effectiveness of health-related interventions [14,19-21]. Therefore, the investigation of UX in the context of digital technologies in patient care is of great importance and should be considered early during the development process [14,15,22]. Thus, a thorough formative evaluation of the UX should precede a summative evaluation to optimize the app from the end user’s perspective [14].

As a multidimensional construct, UX comprises various dimensions that can differ depending on the underlying model, understanding, and context [15,17,19]. According to Thielsch and Salaschek [21], the dimensions *content*, *usability*, and *aesthetics* are particularly important in a research-related context. In addition, *feelings and emotions* that arise through interaction with the technical medium are described as an important aspect of UX [15,16]. As personal data are collected, passed on, and stored in digital care structures, *trust* in the technology is another predictor of future app use [21].

### Behavior Change and Self-Management Apps for Patients With Chronic Skin Diseases

Apps to support behavior change and disease-specific self-management have also been developed and evaluated in the context of chronic skin diseases. These are usually aimed at specific groups, such as parents of children with atopic dermatitis (AD) [23,24]; patients with AD [25,26]; or people with specific areas of the skin affected, such as hand and foot eczema [27]. Weigandt et al [27] developed the first smartphone app in Germany specifically for hand and foot eczema, with which patients can monitor and manage their disease following a patient educational intervention by photographing their skin, tracking their quality of life and symptoms, and chatting with their dermatologist.

However, thus far, such technology does not exist for work-related skin diseases (WRSDs) even though they have been among the most common work-related diseases in Germany for decades [28]. Up to 90% of all diagnosed cases affect the hands in the form of irritant or allergic contact eczema, which often occurs in combination with a genetic predisposition to AD [29-32]. Work-related hand eczema is usually caused by hazardous activities in the workplace (eg, skin contact with allergens and irritants, wet work, high handwashing frequencies, and mechanical stress). Workers in the health care, metalworking, hairdressing, and construction sectors are at a particularly high risk, which results in high prevalence rates [30,31]. Due to the high relevance of WRSDs, the German Social Accident Insurance has developed a complex and hierarchical multistep procedure for patients with WRSDs, with different outpatient and inpatient prevention measures depending on the severity of the skin disease. In the case of severe or recurrent skin diseases, patients are offered to participate in an inpatient interprofessional rehabilitation program with a duration of 3 weeks. In German occupational dermatology, this program is also known as tertiary individual prevention (TIP) [29,33]. An important element of TIP are health educational interventions [34] with the aim of gaining disease-specific knowledge and increasing the motivation to implement and optimize individual skin protection behavior. In addition, individual strategies for skin protection behavior are developed with occupational therapists and practiced as part of a workplace simulation in occupational therapy. To restore the skin barrier and reduce the risk of recurrence of hand eczema, it is important to implement skin protection behavior (eg, regular use of skin protection and skin care products as well as reducing the frequency of handwashing by using mild detergents) in the long term [34-36]. However, changing skin protection behavior is complex and requires a high level of self-management after participating in the TIP as the new behavior must be successfully transferred to and implemented in the professional and private contexts. A structured maintenance program that supports patients in this subsequent implementation of skin protection behavior does not yet exist [34]. To fill this gap, we developed the Mein Hautschutz im Alltag (MiA) app (MiA translates to “My skin protection in everyday life”) to support the self-management of patients with WRSDs after the TIP [37].

### Objectives of This Study

The aim of this study was to evaluate the UX of and adherence to the MiA app and pilot the feasibility of an app-based maintenance program in our clinical setting. In this publication, we report the following results: (1) description of the intervention in a standardized manner; (2) UX, subjective quality, and perceived impact of the MiA app; and (3) adherence to the MiA app.

## Methods

### Overview

The occupational dermatology maintenance program is a complex intervention consisting of several interacting components (Multimedia Appendix 1). According to the Medical Research Council’s framework, the development of complex interventions is based on a 4-stage process consisting of development [37], feasibility, evaluation, and implementation [38]. This study focuses on feasibility (phase 2).

### Intervention

The maintenance program is offered by the Institute for Interdisciplinary Dermatological Prevention and Rehabilitation (iDerm), Osnabrück, Germany, which is a specialized center in Germany for inpatient and outpatient interprofessional treatment of patients with WRSDs.

The maintenance program consists of 2 main elements: *individual goal-setting interview* and the MiA app. The systematic development of these elements is described in detail elsewhere [37].

The *individual goal-setting interview* is conducted during the inpatient stay by a health educator face-to-face 5 or 6 days before discharge. The goals, which are defined by the patients, are entered into the app on the coaching platform by the health educator and can be viewed by patients via the app. During the subsequent onboarding, patients receive an introduction and explanations of the various app functions. Patients are also given access to a video via a QR code that shows the app with screenshots and explains the app’s functions.

**Figure 2:**
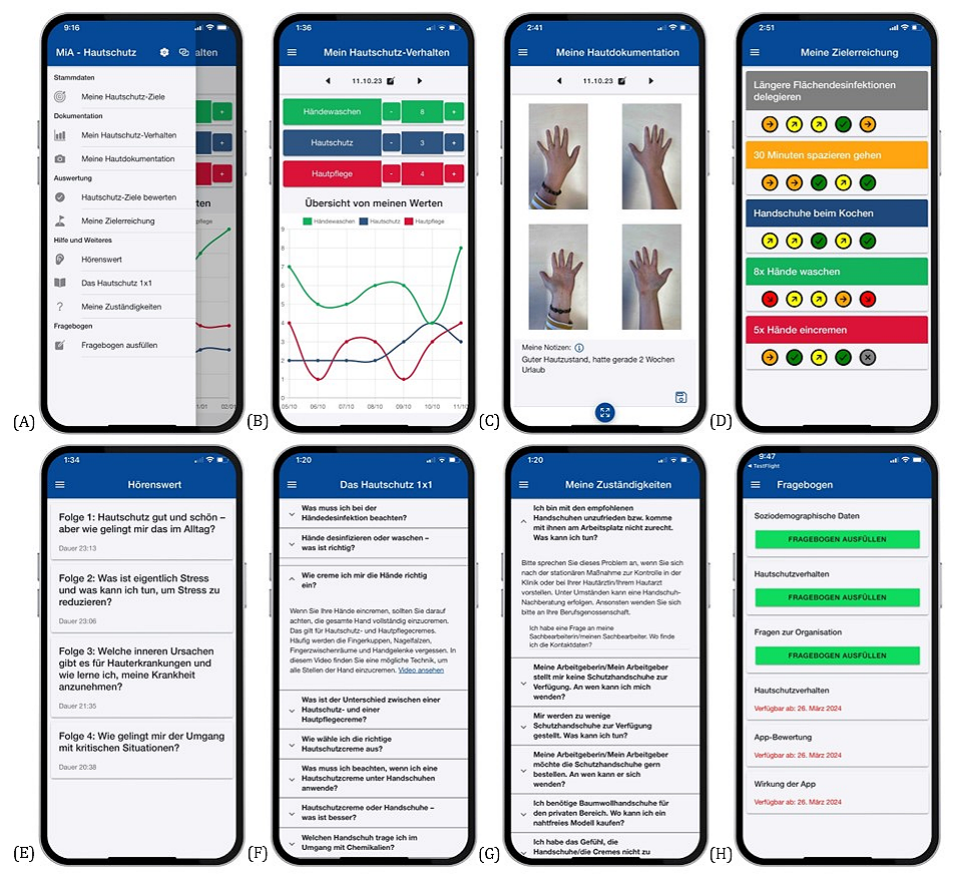
(A) Overview of the menu; (B) overview of the My Skin Protection Behavior component with the option to enter the frequencies of skin cleansing (green line) and applications of skin care cream (red line) and skin protection cream (blue line); (C) overview of the My Skin Documentation component, which allows for taking pictures of the front and back side of the right and left hand that can be enlarged and to which personal notes can be added; (D) overview of the My Skin Protection Goals component with the overview of the goal achievement; (E) To Listen component and 4 podcast episodes; (F) overview of the Skin Protection 101 component with different questions and an example unfolded answer; (G) overview of the My Accountabilities component with different questions and an exemplary unfolded answer; and (H) presentation of the research questionnaire with the 6 categories, whereby categories 1 to 3 (sociodemographic data, skin protection behavior, and questions about the organization) can be completed immediately and categories 4 to 6 (skin protection behavior, app evaluation, and impact of the app) are activated after 12 weeks of use.

MiA is a fully automated app with free access for study participants and consists of 6 components focusing either on interaction or information. The smartphone-based components with interactive nature are named My Skin Protection Goals (German: *Meine Hautschutz-Ziele*), My Skin Protection Behavior (German: *Mein Hautschutz-Verhalten*), and My Skin Documentation (German: *Meine Hautdokumentation*). The smartphone-based components To Listen (German: *Hörenswert*), Skin Protection 101 (German: *Das Hautschutz 1x1*), and My Accountabilities (German: *Meine Zuständigkeiten*) provide different information about the skin disease in different modes of delivery (podcasts, textboxes, and videos; Figure 2). In addition, users automatically receive a reminder message if no information has been entered into the app for a period of 14 days.

Multimedia Appendix 1 provides a more detailed description of each component according to the Template for Intervention Description and Replication checklist [39,40], including what, why, how often, and when components are unlocked during the maintenance program and where and which tailoring options are available. Table 1 describes the contents of the active components of the intervention in a standardized manner using the Behavior Change Technique Taxonomy version 1 [7,41].

**Table 1:** Behavior change techniques (BCTs) in the 12-week occupational dermatology maintenance program for improving health behavior change in patients with work-related skin diseases based on the BCT Taxonomy version 1 [7,41].

Component			Description		BCTs
**Face-to-face intervention**					
	Individual goal-setting interview	Formulation of individual skin protection goals		1.1: goal setting (behavior)	
**MiA^a^ app**					
	My Skin Protection Goals	Monitoring of individual skin protection goals and assessment of their achievement		1.5: review behavior goals	
	My Skin Protection Goals	Visualization of goal achievement over time using arrows		1.6: discrepancy between current behavior and goal	
	My Skin Protection Goals	Motivating feedback messages depending on the evaluation of the goal		3.1: social support (unspecified)	
	My Skin Protection Behavior	Tracking of skin protection behavior and monitoring the progress		2.3: self-monitoring of behavior	
	My Skin Documentation	Recording and observing the skin condition		2.5: monitoring of outcomes of behavior without feedback	
	To Listen	Information about the skin disease, associated difficulties, and strategies		Episode 1: 4.1—instruction on how to perform the behavior and 15.1—verbal persuasion about capability Episode 2: 5.1—information about health consequences, 5.2—salience of consequences, 8.2—behavior substitution, 12.4—distraction, and 15.1—verbal persuasion about capability Episode 3: 8.2—behavior substitution, 5.1—information about health consequences, 5.2—salience of consequences, 4.2—information about antecedents, and 15.1—verbal persuasion about capability Episode 4: 3.1—social support (unspecified), 3.3—social support (emotional), and 15.1—verbal persuasion about capability	
	Skin Protection 101	Information about seminar content on skin protection, itching, and stress		4.1: instruction on how to perform the behavior 5.1: information about health consequences	
	My Accountabilities	Information about organizational issues related to care and responsibilities		5.3: inform about social and environmental consequences	

^a^MiA: Mein Hautschutz im Alltag (My skin protection in everyday life).

### Ethical Considerations

This study was approved by the Ethics Committee of Osnabrück University (Ethics-50/2022 and Ethics-15/2023). All patients assessed for eligibility were informed verbally and in writing about the study and its voluntary nature. All participants had to sign a declaration of consent to take part in the study. The participants were informed that data collection would be pseudonymized and that the evaluation and publication of the results would be anonymous. They received no compensation for their participation.

### Inclusion Criteria and Study Design

Recruitment took place at iDerm in Osnabrück on the fourth day during the TIP. Study information was provided in patient groups as 7 to 9 new patients are usually admitted to the TIP each week. After all patients had been fully informed about the study, they were given sufficient time to decide whether to participate. Subsequently, interested patients received the written study information and declaration of consent. The inclusion criteria were (1) signed declaration of consent for study participation; (2) legal age (>18 years); (3) sufficient German language skills to understand the app content, participate in focus groups, and complete questionnaires; and (4) access to an internet-enabled smartphone (for step 2).

**Figure 3:**
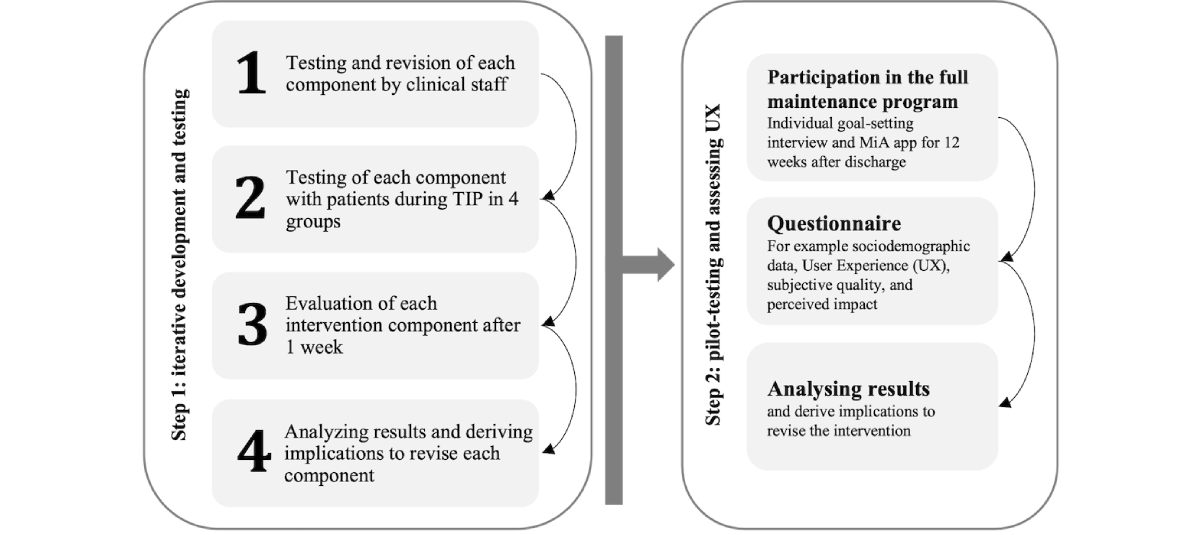
Study process of multistage iterative testing of the app-based maintenance program for patients with work-related skin diseases consisting of (1) iterative development and testing and (2) 12-week testing and piloting. MiA: Mein Hautschutz im Alltag (My skin protection in everyday life); TIP: tertiary individual prevention; UX: user experience.

This study followed a user-centered and multistage iterative approach, which is a well-known and widespread procedure in the development of technologies in health care and UX research [42,43]. This approach allows for the consideration of the opinions and feedback of the target group as future users of the app in a participatory manner [23,44-46]. As illustrated in Figure 3, the entire process consisted of 2 steps with both qualitative and quantitative data collection.

### Instrument

Various instruments have been developed to assess the UX of mobile health apps. Among them, the user version of the Mobile Application Rating Scale (uMARS) is a well-known and reliable instrument that measures the UX of health apps from the user’s perspective [47]. The uMARS is based on the Mobile Application Rating Scale, which is used to assess the quality of apps by clinical or technical experts [48,49]. The uMARS is made up of 16 items and comprises the UX dimensions of *engagement*, *functionality*, *aesthetics*, and *information quality*. The scales of subjective quality and perceived impact assess additional information about recommendation; willingness to use and pay for the app; and information regarding awareness and knowledge of and intention for behavior change. For this study, an instrument for step 1 and step 2 was developed based on the uMARS and the model shown in Figure 1.

Statistical analyses of the quantitative data were carried out using SPSS (version 28; IBM Corp). We calculated a score for all 5 UX dimensions as well as a score for all UX items. Adherence to the My Skin Protection Behavior, My Skin Documentation, and My Goal Achievement functions was determined based on the adherence values in Multimedia Appendix 1 and by dichotomizing the data into *adherent* and *nonadherent*. For continuous data (eg, age and UX), we calculated the mean, median, IQR, and SD. We present categorical data (eg, gender, subjective quality, and perceived impact) in frequencies and percentages.

### Step 1: Iterative Development and Testing of Intervention Components

#### Overview

In the first step, the maintenance program was tested by 4 different patient groups during participation in the TIP at the iDerm in Osnabrück between November 2022 and November 2023. Patients were recruited on the fourth day of the TIP with a subsequent test phase of 2 weeks. During the test phase, patients were asked to document their overall impression as well as positive aspects and aspects requiring improvement of the tested component in a standardized documentation form. The documentation forms were not evaluated by the researchers as they merely served as preparation for the patients themselves for the focus group discussions. Each group tested and evaluated specific maintenance components as follows: group 1 evaluated *To listen*, group 2 evaluated the *face-to-face goal setting interview*, group 3 evaluated My Skin Protection Behavior and My Accountabilities on the app, and group 4 evaluated Skin Protection 101 and My Skin Documentation on the app.

#### Methods of Data Collection in Step 1

After the test phase, the patients completed a short quantitative questionnaire that assessed their overall impression of the maintenance components tested using selected items from the uMARS. The focus of step 1 was qualitative data collected via 4 subsequent focus group discussions. The aim of the focus group discussions was to record positive and negative aspects of the tested components, identify problems and challenges regarding their use, and jointly develop opportunities for improvements.

The focus group discussions were conducted by 2 moderators (NR and ML) based on guiding questions regarding content-related, methodological, and technical aspects. The guiding questions served as orientation. Additions to or deepening of the questions and topics that arose in the course of the discussions were permitted. The interviews were recorded using an audio recorder (Olympus LS-P4 linear pulse-code modulation recorder). Knowledge mapping was chosen for data analysis as it allows for the combination of data collection and data processing [50]. The results were written down in keywords on moderation cards and clustered on a metaplan board. The participants validated the results for completeness and correctness for each topic. In addition, experiences from the test phase and key results were documented by a research assistant in a protocol. Aspects of the components that needed to be improved were then revised in the next iteration.

### Step 2: Pilot-Testing and Assessing the UX

#### Overview

In the second step, the entire maintenance program, consisting of the face-to-face goal-setting interview and the MiA app with all its components, was tested by patients and formatively evaluated. Recruitment took place over a period of 10 weeks from January 2024 to March 2024. Patients who voluntarily agreed to participate were then given an appointment for the goal-setting interview as a one-to-one meeting and the subsequent onboarding to the app in the third week. The app was tested over a period of 12 weeks after discharge from the TIP, with 2 additional weeks to fill out the second questionnaire. Patients who did not complete the questionnaire within these 2 weeks received a reminder in the form of a paper questionnaire with a prepaid return envelope by post.

#### Methods of Data Collection in Step 2

##### Overview

The evaluation was carried out using a quantitative questionnaire consisting of 6 blocks. Blocks 1 to 3 were available immediately after registration, and blocks 4 to 6 were activated after the maintenance period of 12 weeks after discharge. In total, the instrument consisted of 85 questions about sociodemographic data; skin protection behavior before and after the rehabilitation program; and evaluation of the UX, subjective quality, and perceived effects, as well as questions about the structural, process, and outcome quality of the intervention.

In addition to closed-ended questions, the questionnaire also included open-ended questions to allow the participants to provide further details about the individual functions beyond the closed-ended questions [15]. The comprehensibility of the questions and the time required for completion were determined in a pretest with 16 patients before the study. In this publication, we report the results on sociodemographic data, UX evaluation, subjective quality, and perceived impact of the intervention.

##### UX Evaluation

To assess the UX, the relevant items of the uMARS were assigned to the UX dimensions shown in Figure 1 and supplemented with additional aspects as required. We measured feelings and emotions using 5 items (eg, “I find the app entertaining” or “The app offers me enough options to customise it to my personal requirements and needs”), content using 4 items (eg, “Is the content of the app relevant to you?” or “Are the texts understandable?”), usability using 6 items (eg, “How well and quickly do the app’s applications [buttons, menus] respond?” or “How easy was it for you to learn how to use the app?”), aesthetic using 2 items (“How high are the quality and resolution of the images and texts?” and “How would you rate the overall appearance of the app?”), and trust using 3 items (eg, “The information in the app appears to come from a credible source” and “Are you concerned that data you have entered into the app could be uploaded to third parties?”). All questions were answered on 5-point Likert scales that differed in their content depending on the dimension and item.

##### Subjective Quality and Perceived Impact

Both aspects were assessed using the uMARS [47]. Subjective quality was measured using the items related to recommendation, willingness to use in the following 12 months, and willingness to pay and overall rating by stars.

The perceived impact scale comprised 5 closed-ended questions on awareness and knowledge of and motivation for skin protection behavior; encouragement to seek for support; and enabling skin protection behavior on a 5-point Likert scale and a supplementary open-ended question on which functions were particularly helpful for implementing skin protection behavior.

##### Adherence

An important result for this study was the analysis of the frequency of use of individual components as this represented a central criterion for adherence to the intervention. We used a simple definition according to Donkin et al [51] and understood adherence as “the degree to which the user followed the programme as it was designed” [51]. This definition has already been applied in the context of other digital health interventions [45]. In this study, adherence was determined by the frequency with which the skin protection behavior was entered, how often photos were taken, and how often the goals were assessed. Adherence was high if the component My Skin Protection Behavior was used daily and the functions My Skin Documentation and My Skin Protection Goals were used weekly.

## Results

### Step 1

#### Study Participants

A total of 23 patients took part in the testing of the intervention components in step 1, of whom 10 (43%) were female and 13 (57%) were male. The average age of the participants was 51 (SD 11; range 27-62) years.

The patients worked in health care (8/23, 35%), the metalwork industry (4/23, 17%), hairdressing (3/23, 13%), construction (2/23, 9%), or other professions (6/23, 26%).

#### Main Results

The results of step 1 were used for iterative development to further optimize the intervention for phase 2. For example, in the My Skin Protection Behavior function, the scale of the days and frequencies in the diagram was too small. This was adjusted and enlarged in the revision. In the My Skin Documentation function, every entry was automatically saved in the comment function. This always hindered further input for a few seconds. A button was added so that users could save their comments at the end by themselves. Patients also highlighted that the app had a simple structure and reminded them of apps they already knew, had a good usability and visual presentation, and was factual without playful elements. A more detailed presentation of the results of step 1 and the associated modifications for step 2 can be found in Multimedia Appendix 2.

### Step 2

#### Study Participants

**Figure 4:**
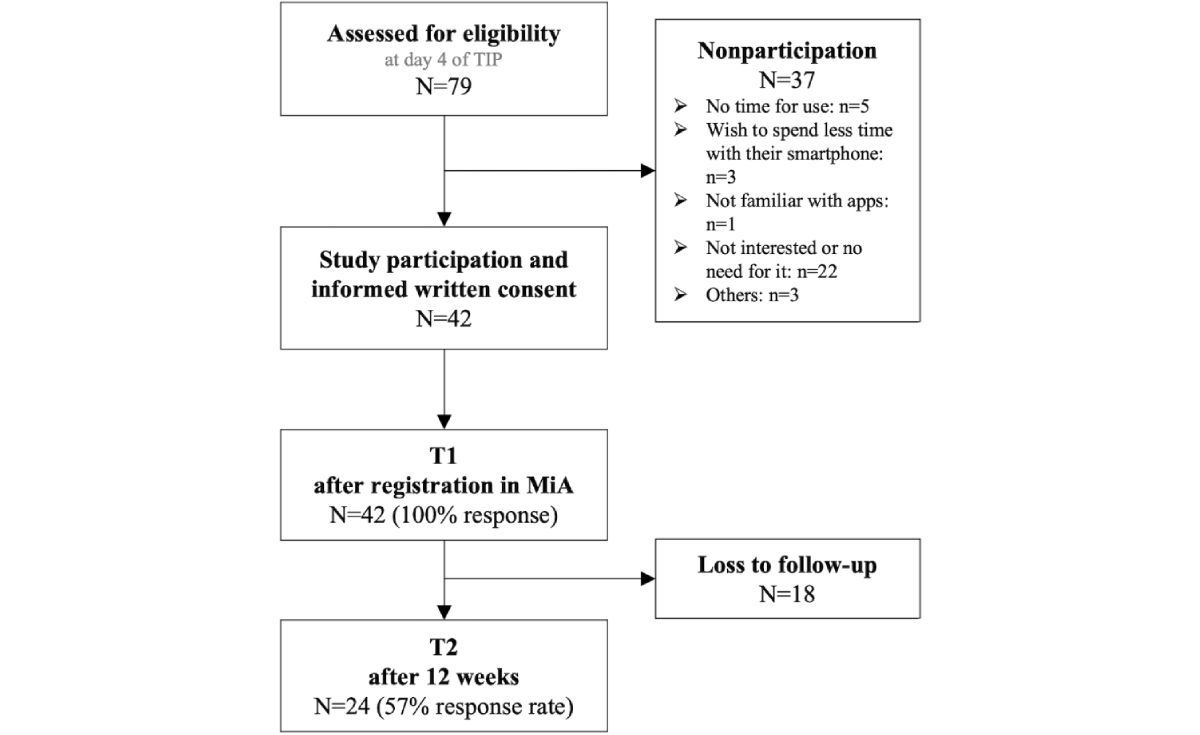
Flowchart of the study population in step 2, including assessment for eligibility, participation, and follow-up at T1 and T2. MiA: Mein Hautschutz im Alltag (My skin protection in everyday life); TIP: tertiary individual prevention.

Over 10 weeks (study period for step 2), a total of 79 patients participated in the TIP program, of whom 42 (53%) agreed to take part in the intervention and gave informed consent for study participation. The main reasons for nonparticipation were no interest or no subjectively felt need for the intervention (Figure 4).

All 42 patients took part in the individual goal-setting interview and received access to the MiA app for the 12-week testing phase. At T1, all participants completed the questionnaires (42/42, 100% response rate), and at T2, the response rate was 57% (24/42). Table 2 summarizes the sociodemographic data of the patients enrolled in this study.

**Table 2:** Sociodemographic data of the study participants at T1 (N=42).

Sociodemographics			Values
**Sex, n (%)**			
	Male	24 (57)	
	Female	18 (43)	
**Age (y)**			
	Values, mean (SD)	49.5 (13.1)	
	Values, median (IQR)	55.0 (18.6)	
**Occupational categories, n (%)**			
	Health care professions	17 (40)	
	Metalwork industry	9 (21)	
	Construction	5 (12)	
	Hairdressing	1 (2)	
	Food processing	2 (5)	
	Other	5 (12)	
	Pensioner	1 (2)	
	Currently unemployed	2 (5)	
**Highest educational level, n (%)**			
	Secondary school or elementary school–leaving certificate	9 (21)	
	Intermediate school–leaving certificate or secondary school–leaving certificate	15 (36)	
	“Meister”^a^	3 (7)	
	College certificate	5 (12)	
	General higher education entrance qualification or A-levels	7 (17)	
	Bachelor’s degree	1 (2)	
	Master’s degree or diploma	2 (5)	
**Employment status, n (%)**			
	Self-employed	4 (10)	
	Employed	35 (83)	
	Unemployed	3 (7)	
**Partnership, n (%)**			
	Yes	32 (76)	
	No	10 (24)	

^a^Person with a higher vocational qualification in a craft, also known as “master craftsman” or “master craftswoman.”

#### UX Results

**Figure 5:**
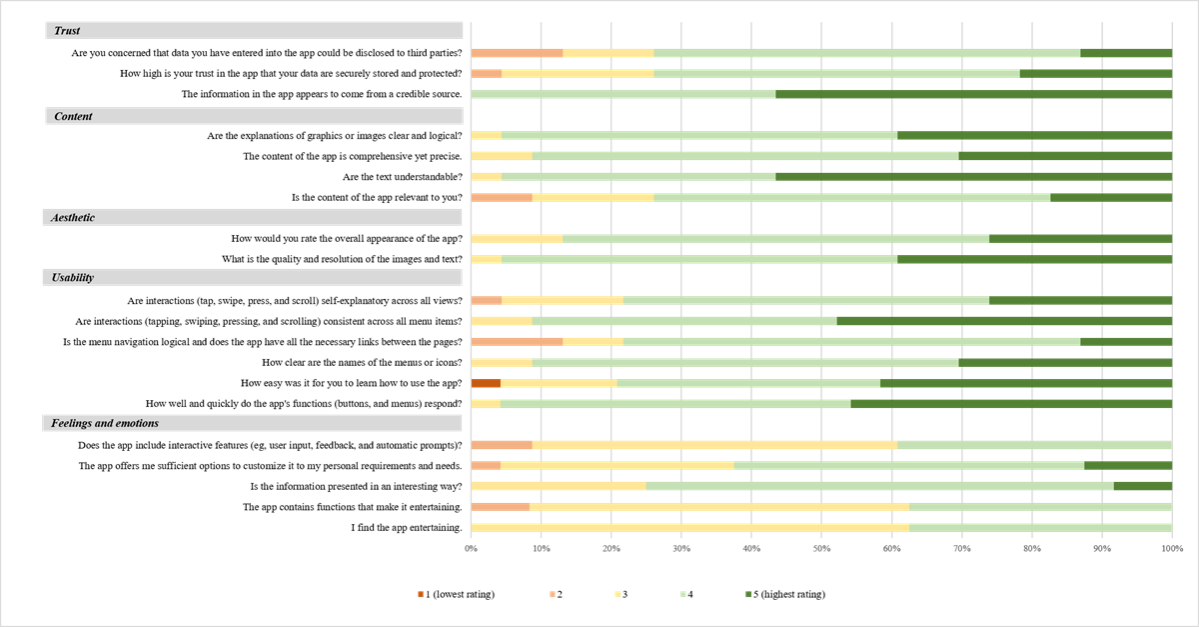
Results for the 5 user experience (UX) dimensions—content, usability, aesthetic, trust, and feelings and emotions—from the participants at T2 (n=24). The ratings range from 1 to 5. A rating of 1 means a low rating, and a rating of 5 is a high rating.

The average UX score, with a theoretical minimum of 20 and a theoretical maximum of 100, was 80.18 (SD 8.94). Figure 5 shows the results for the assessment of the UX from the *trust*, *content*, *aesthetic*, *usability*, and *feelings and emotions* dimensions. The raw data for these items can be found in Multimedia Appendix 3. The results show overall positive ratings (scores of 4 and 5). The *feelings and emotions* dimension stood out, with comparatively frequent medium ratings and no ratings of 5 on 60% (3/5) of the items. Furthermore, the item “How easy was it for you to learn how to use the app?” in the *usability* dimension was the only item with a score of 1 by 2% (1/42) of the participants.

#### Subjective Quality and Perceived Impact

Overall, the results showed a high subjective quality (Multimedia Appendix 4). Approximately 75% (18/24) of the patients would recommend the app to others. The average willingness to use the app in the following 12 months was “once a week” (7/25, 30%). Most (14/24, 58%) were not willing to pay for the app. The app was most frequently rated with 4 out of 5 stars (15/24, 65%).

**Figure 6:**
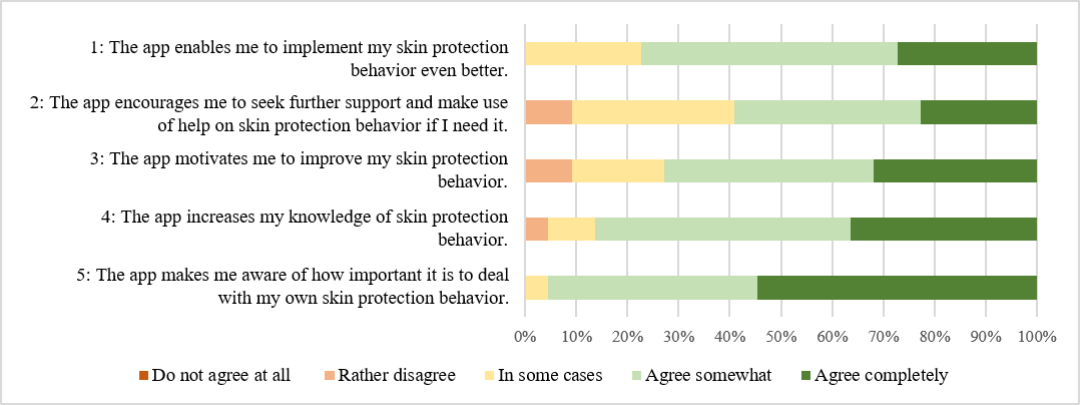
Results on the perceived impact of the Mein Hautschutz im Alltag (My skin protection in everyday life) app regarding skin protection behavior by the participants at T2 (n=24).

As shown in Figure 6, there was an average high level of agreement regarding the subjective impact of the MiA app in relation to skin protection behavior and important determinants (eg, awareness, motivation, or knowledge). All items were rated by more than half (Item 1: 17/24, 71%; Item 2: 13/24, 54%; Item 3: 16/24, 67%; Item 4: 19/24, 79%; Item 5: 21/24, 88%) of the participants as “somewhat agree” or “completely agree” (Multimedia Appendix 4).

#### Adherence

A total of 135 goals were formulated, which corresponds to an average of 3.2 (SD 1.46) goals per person (Table 3). Textbox 1 contains exemplary goals from the goal-setting interviews. Of the 42 participants, 1 (2%) did not formulate any goals, and 10 (24%) formulated the maximum of 5 goals. Most of the goals related to the skin protection category.

**Table 3:** Number and categories of goals formulated in the individual goal-setting interview by the patients as part of the maintenance program during tertiary individual prevention (N=135).

	Skin cleansing, n (%)	Skin protection, n (%)	Skin care, n (%)	Social support, n (%)	Other, n (%)	Total, n (%)
Goal 1	8 (5.9)	12 (8.9)	11 (8.1)	5 (3.7)	5 (3.7)	41 (30.4)
Goal 2	5 (3.7)	13 (9.6)	5 (3.7)	2 (1.5)	10 (7.4)	35 (25.9)
Goal 3	3 (2.2)	9 (6.7)	7 (5.2)	4 (3)	6 (4.4)	29 (21.5)
Goal 4	4 (3)	8 (5.9)	4 (3)	3 (2.2)	1 (0.7)	20 (14.8)
Goal 5	3 (2.2)	4 (3)	0 (0)	2 (1.5)	1 (0.7)	10 (7.4)
Total	23 (17)	46 (34.1)	27 (20)	16 (11.9)	23 (17)	135 (100)

**Example goals set by the patients in the individual goal-setting interview.**

**Skin cleansing**

- I intend to wash my hands less during the day.
- I intend to reduce the water temperature when cleansing my skin.
- I intend to wash my hands a maximum of 10 times a day.

**Skin protection**

- I will put on cotton gloves after applying cream.
- I will consistently wear padded gloves for activities involving mechanical pressure on the palms of my hands.
- I intend to apply a skin protection cream at least 3 times a day.

**Skin care**

- I will apply a skin care cream right at the end of the working day.
- I intend to apply a skin care product to my hands 5 times a day.
- I will apply cream to my whole body once a day (in the evening).

**Social support**

- I will use a cell phone alarm to remind me to apply skin care cream every day.
- I will ask for support at work (e.g. if the skin condition is bad).

**Other**

- I intend to pay more attention to myself and say “no” more often.
- I'm going to yoga class every Tuesday evening.
- I intend to go for a walk 2-3 times a week.

**Figure 7:**
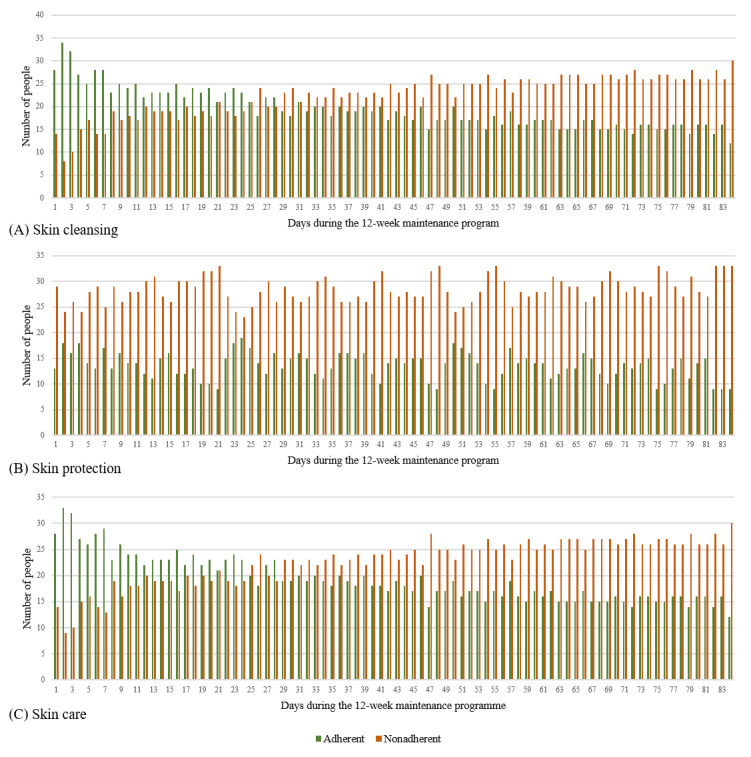
(A) Adherence to tracking of skin cleansing on the app function My Skin Protection Behavior, (B) adherence to tracking of skin protection on the app function My Skin Protection Behavior, and (C) adherence to tracking of skin care on the app function My Skin Protection Behavior by all participants during the 12-week pilot-testing (n=42).

The results in terms of adherence to the app function My skin protection behavior are shown in Figure 7. The raw data can be found in Multimedia Appendix 5. The results show differences in adherence between the dimensions of skin protection behavior at the beginning of the maintenance program. On day 1, there were significantly more adherent than nonadherent participants to the dimensions of skin cleansing (adherent participants: 28/42, 67%; nonadherent participants: 14/42, 33%; *P*=.04) and skin care (adherent participants: 28/42, 67%; nonadherent participants: 14/42, 33%; *P*=.04) compared to the skin protection dimension, which had significantly more nonadherent than adherent participants (adherent participants: 13/42, 31%; nonadherent participants: 29/42, 69%; *P*=.02).

Furthermore, there was a difference in adherence over time during the 12-week maintenance program. As shown in Figures 7A and 7C, the number of nonadherent participants to the skin cleansing and skin care dimensions was higher than the number of adherent participants from day 26 onward. After 12 weeks, there were significantly more nonadherent than adherent participants who tracked their skin protection behavior daily in all 3 dimensions: skin cleansing (adherent participants: 12/42, 29%; nonadherent participants: 30/42, 71%; *P*=.008), skin care (adherent participants: 12/42, 29%; nonadherent participants: 30/42, 71%; *P*=.008), and skin protection (adherent participants: 9/42, 21%; nonadherent participants: 33/42, 79%; *P*<.001; Figures 7A, 7B, and 7C).

**Figure 8:**
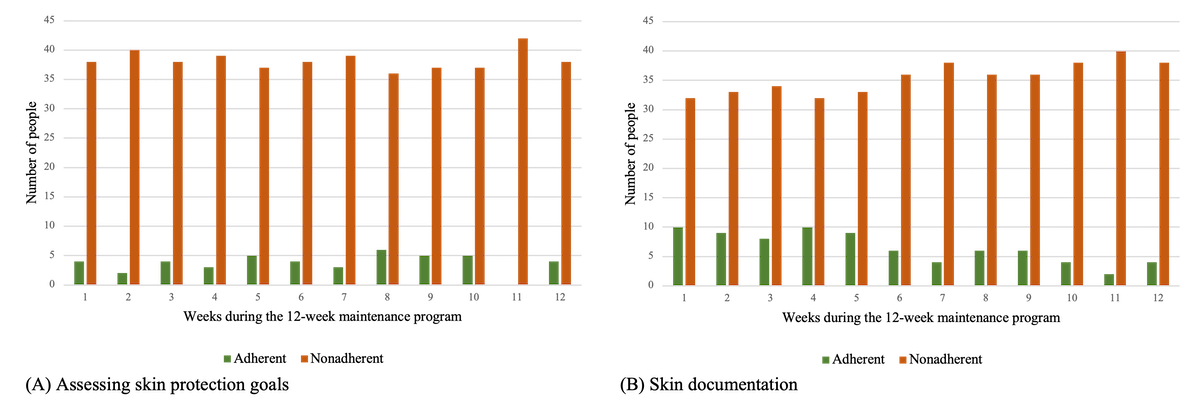
(A) Adherence to the achievement of skin protection goals on the app function My Skin Protection Goals and (B) adherence to skin documentation on the app function My Skin Documentation by all participants during the 12-week pilot-testing (n=42).

Adherence to rating the achievement of skin protection goals (Figure 8A) and photo documentation (Figure 8B) was lower than adherence to the tracking of skin protection behavior (Multimedia Appendix 5). The number of nonadherent participants was clearly lower than the number of adherent participants in each week of the 12-week maintenance program for both app functions. At the beginning, in week 1 (adherent participants: 4/42, 10%; nonadherent participants: 38/42, 90%; *P*<.001), and at the end, in week 12 (adherent participants: 4/42, 10%; nonadherent participants: 38/42, 90%; *P*<.001), there were significantly more nonadherent than adherent participants who assessed their skin protection goals. Similar results were also found for the app function My Skin Documentation in week 1 (adherent participants: 10/42, 24%; nonadherent participants: 32/42, 76%; *P*<.001) and week 12 (adherent participants: 4/42, 10%; nonadherent participants: 38/42, 90%; *P*<.001).

## Discussion

### Principal Findings and Comparison With Prior Work

The aim of this study was to evaluate the UX of and adherence to an app-based maintenance program. The MiA app is a self-management tool for patients with WRSDs after participating in an inpatient rehabilitation program (TIP) to support change in and maintenance of skin protection behavior. To the best of our knowledge, the MiA app is the first app for people with WRSDs. Our study was conducted by following a multistage and iterative development and pilot-testing process, with this paper focusing on the results of phase 2.

UX research is an important part in the development of apps to identify and address potential barriers to the use of apps as early as possible. It should precede the summative evaluation [38]. However, many studies investigating the influence of app-based interventions in the dermatological setting only report on clinical outcomes such as quality of life, eczema severity, or itching [24,27] or app use behavior [25,26]. Zvulunov et al [24] point out that there are missing data on the usability of and adherence to apps in the field of chronic skin diseases.

We evaluated the UX using the dimensions of *feelings and emotions*, *usability*, *aesthetics*, *content*, and *trust*. Our study generally showed high to very high ratings for all dimensions with the exception of *feelings and emotions*, which was rated comparatively lower. Studies show that gamified apps can improve the interaction, motivation, and adherence [23,52]. However, the benefit of gamification differs between genders and decreases with age, making it difficult to successfully implement those functions in our heterogeneous study population [53]. According to Tolks et al [53], gamified apps are particularly useful for people with low motivation to implement a behavior, and Matterne et al [54] were able to show that patients with WRSDs have a high motivation to implement skin protection, which increased significantly during the TIP. Furthermore, in a focus group discussion in our study, patients expressed that the app should not contain any playful elements and should remain factual as it dealt with a disease. The extent to which the app is used also depends on the app’s BCTs. Milne-Ives et al [55] were able to demonstrate that some BCTs are associated with higher use of and engagement with an app. These included goal setting and feedback, self-monitoring, and social support, which are also part of the MiA app. However, in the future, the app could be expanded to include further interactive functions that allow users, for example, to assess their skin condition more accurately. One possibility might be to analyze the severity of hand eczema in photo documentation using artificial intelligence [24]. Weigandt et al [27] were able to show that the eczema severity determined via photos significantly correlates with live assessment by dermatologists. Therefore, photographs appear to allow for reliable results regarding the actual severity of hand eczema.

The presentation of information on the app was rated as particularly interesting by patients. We attribute this to the different ways in which the information was presented (podcasts, videos, and short questions and answers).

The descriptive results of the UX also show single exceptions regarding usability. Due to the small study cohort at T2, we were unable to test any associations between UX and the sociodemographic characteristics of the participants. However, digital health literacy plays an important role in usability. It is known that, in addition to app-specific factors, the use of an app may also depend on personal factors such as age, sex, educational level, and socioeconomic status [15,27,56]. Nationally and internationally, it has been shown that older people with a lower educational level and of a lower socioeconomic status tend to have a lower digital health literacy [57,58]. With an average age of 49.5 (SD 13.1) years and a high proportion of people with elementary and secondary school–leaving certification (24/42, 57%), our study cohort had characteristics that could indicate a lower level of digital health literacy. Therefore, a potential influence of digital health literacy on the use and handling of the MiA app is conceivable. Further research should be carried out to determine which socioeconomic characteristics in our field of application are particularly affected by low digital health literacy. This is important to provide patients with individual and tailored support from health care staff during the onboarding process [59]. Nevertheless, onboarding via personal contact in face-to-face meetings is a particular strength of our study because we were able to respond to the individual digital competence of the patients.

We found differences in adherence between the interactive functions of the app. The daily tracking of skin protection behavior was used more often than the weekly skin documentation and goal assessment. Furthermore, skin care and skin cleansing were tracked more frequently than skin protection. In addition, adherence to skin cleansing and skin care tracking decreased over the 12 weeks. The lower tracking of skin protection at the beginning might be explained by the fact that patients are at home for a period of 3 weeks after discharge and do not start working in their profession straight away. That means that the use of skin protection creams at work only becomes relevant from day 21 onward. The digital documentation of skin protection creams may not have increased from day 21 onward as the practical implementation of digital tracking during work is not feasible. However, in contrast to this, we observed a clearly higher proportion of formulated individual goals in the skin protection category than in the skin cleansing, skin care, support, and other categories. This emphasizes that the topic of skin protection is nevertheless an important aspect for patients after the TIP even if this cannot be reflected in skin protection tracking for the aforementioned reasons. Against this background, linking both functions appears to be useful to adequately cover all dimensions of skin protection behavior. There are various possible reasons for the less frequent use of weekly photo documentation and target assessment. First, both components are technically more complex and more challenging functions and require more time. One possible explanation for the low level of photo documentation could be that photos are not taken routinely by the users but, rather, when acute skin changes occur. The weekly rhythm we set may not correlate with the progression of the individual skin diseases. WRSDs occur in over half of cases with a comorbidity of AD and progress in intervals of varying length [33]. The goal assessment is also cognitively more complex because it requires reflection on the last behavior performed by the users and the decision of the appropriate response option. The reasons for the less frequent use of both functions should be further investigated, for example, by users and nonusers reporting on their experiences, motivation, and the usefulness of these functions (eg, in qualitative interviews [56]). Nevertheless, it should be kept in mind that high adherence to the app functions (eg, skin protection tracking) is not necessarily associated with behavior changes and better clinical outcomes. In everyday life, skin diseases are associated with a high level of adherence to therapy. Daily documentation may be too time-consuming to implement in practice, meaning that regular app use does not necessarily lead to better outcomes in terms of eczema severity or quality of life [27]. The average willingness to use the app approximately once a week supports this assumption. The use of the MiA app after the TIP can also be individual depending on the extent to which patients benefited from patient education during the TIP. Another influencing factor on app use could be the duration of the disease. TIP patients have usually had the skin disease for several years and have developed their own self-management strategies in the past. In the future, it could be investigated what are the use and benefits for patients with milder forms of WRSDs (eg, as part of outpatient care). The results on the perceived impact show that the MiA app can also be used as a tool for disseminating knowledge about skin protection–related content.

Overall, our results are consistent with those of other studies on dermatological apps and, thus, show expected results on adherence, which decreases over time [25]. Shah et al [26] showed that the average duration of use of an app for people with AD was approximately 6 weeks. The frequent short duration of use of app-based interventions and the associated lack of evidence of long-term effects is a general and well-known problem in this research context [2,26].

We were able to demonstrate a high quality of the app. Despite the low adherence, the results on the perceived effect also show promising indications that the app might influence relevant determinants such as motivation, knowledge, or awareness regarding the change in health behavior according to the subjective statements of the participants. However, the effect of the MiA app on skin protection behavior, quality of life, and eczema severity remains unclear thus far and should be further investigated in the future in comparison with a control group.

### Limitations

In addition to the limitations already mentioned, this study has further limitations that should be considered when interpreting the results. First, UX is a multifactorial and complex construct. There are different methods for assessing UX that have their own strengths and limitations [13]. While questionnaires are cost-efficient and provide quick results, dishonest answers and individual interpretations of the results can be disadvantageous. Focus group discussions are easy to conduct but dependent on the interviewing skills of those conducting them [14]. To measure UX, we used a validated instrument as a basis and adapted it to the context and target group of the app. We concede that the validity of the instrument and a direct comparison of the results with those of other studies is possible to a limited extent. The standardization of the definitions of the UX dimensions and the development of validated instruments for UX research is an important future research focus [19].

The participants in our study matched the sociodemographic characteristics of those of other studies on TIP [60]. Nevertheless, due to the small number of participants, the low use behavior, and a remaining cohort of 57% (24/42) at T2, it was not possible to calculate differences in UX and adherence among age, sex, and educational level.

The target group participated during the development and testing of the MiA app. Nevertheless, their involvement could have been even stronger in the form of agile co-design (eg, for prototyping the app before programming and technical implementation and for identifying realistic adherence [23,61-63]). However, such an approach was not possible due to the organizational processes and duration of this study.

We were also unable to determine the extent to which the informative functions (podcasts, questions and answers, and videos) were used. On the basis of other studies, it can be assumed that the use of educational elements also decreases after a few weeks [25]. However, podcasts activated over the course of the maintenance program could be a way to maintain use over time.

### Conclusions

Our study shows a high UX of the MiA app in almost all dimensions. To increase interaction, functions can be added to the app (eg, use of artificial intelligence with photo documentation to allow for a clearer assessment of the skin condition). We refrained from using gamification due to the heterogeneous study population of patients with WRSDs. We also observed single exceptions in usability. Usability depends, among other things, on the digital health literacy of the user. Future research should investigate in this context which sociodemographic characteristics show lower usability to provide these groups with further support when using the app.

We obtained expected results in terms of adherence, with decreasing use over time, which corresponds to the current state of research in this field. Particularly after resuming work after 3 weeks, daily documentation of skin protection behavior appears to be too time-consuming. The effectiveness of the MiA app in terms of skin protection behavior, quality of life, and eczema severity should be further investigated with a control group.

## Data Availability

The datasets generated or analyzed during this study are available from the corresponding author on reasonable request.

## Appendix

Multimedia Appendix 1 Intervention description.

Multimedia Appendix 2 Results of step 1.

Multimedia Appendix 3 User experience.

Multimedia Appendix 4 Subjective quality and perceived impact.

Multimedia Appendix 5 Adherence to the app’s My skin protection behavior, My skin documentation, and My skin protection goals functions.

## Abbreviations

AD: atopic dermatitis
BCT: behavior change technique
iDerm: Institute for Interdisciplinary Dermatological Prevention and Rehabilitation
MiA: Mein Hautschutz im Alltag (“My skin protection in everyday life”)
TIP: tertiary individual prevention
uMARS: user version of the Mobile Application Rating Scale
UX: user experience
WRSD: work-related skin disease
