# Supplementary material for: User Experience of and Adherence to a Smartphone App to Maintain Behavior Change and Self-Management in Patients With Work-Related Skin Diseases: Multistep, Single-Arm Feasibility Study
Source: JMIR Form Res. 2025 Apr 18;9:e66791. doi: 10.2196/66791 (PMC12048786; doi:10.2196/66791)
Supplement: Multimedia Appendix 1 [file formative_v9i1e66791_app1.docx]

# Multimedia Appendix 1: Intervention description

**Table 1.1** Description of the 12-week occupational dermatology maintenance program after tertiary individual prevention for patients with work-related skin diseases based on the Template for Intervention Description and Replication checklist [39,40].

| **How?**  **Mode of delivery and what?** | | | **Why?**  **Rationale and aims** | **Where?** | **When and how often?** | **Tailoring** |
| --- | --- | --- | --- | --- | --- | --- |
| Component | | Materials and processes |  |  |  |  |
| **Face-to-face intervention** | | | | | | |
|  | Individual goal-setting interview | - Patients define up to 5 individual skin protection goals in one or more of the following categories: skin protection, skin care, skin cleansing, social support, or other. - The starting point is the individual need for optimization from the individual patient’s perspective. - The SMART^a^ rule is taken into account by the health educator for formulating the goals. | Formulation of individual skin protection goals | In the clinic | Once in the third week of inpatient rehabilitation for approximately 30 minutes with a health educator | The number (1-5) and content of the goals are developed and elaborated on individually with the patients. |
|  | Onboarding | - Patients receive an individual access code to the MiA^b^ app. - The elements and functions of the app are demonstrated. - Patients receive access via a QR code for the explanatory video. | Registration and introduction to the MiA app | In the clinic | Once after the individual goal-setting interview for approximately 15 minutes with a health educator | The scope of the introduction is tailored to the digital competence of the patient. |
| **MiA app** | | | | | | |
|  | My skin protection goals (German: Meine Hautschutz-Ziele) | - The following response options are available for the assessment: (1) “I have moved away from my goal,” (2) “Nothing has changed,” (3) “I have come closer to my goal,” (4) “I have achieved my goal,” and (5) “I was unable to pursue my goal this week.” - The answers are displayed graphically in an overview. - Depending on the answer, users receive automatic feedback from a feedback pool that is intended to praise or motivate. | Monitoring of individual skin protection goals and assessment of their achievement | At home during the maintenance program | The assessment of goals can take place every day. Users receive a push notification once a week on Friday reminding them of the target assessment. | The feedback is based on the user’s assessment. The push notification is sent at an individually selected time. |
|  | My skin protection behavior (German: Mein Hautschutz-Verhalten) | - The “+” and “–” buttons can be used to enter the frequency of application of skin protection cream and skin care cream, as well as the number of daily handwashing procedures. - The results are visualized in a diagram. | Daily tracking of skin protection behavior and monitoring of the progress | At home during the maintenance program | The behavior can be entered daily. Users are reminded of the behavior entry daily in weeks 1-2, weekly in weeks 3-6, and every 2 weeks on Fridays from week 7 onward via a push notification. | The push notification is sent at an individually selected time. |
|  | My skin documentation (German: Meine Hautschutzdoku-mentation) | - A photo can be taken of the left back side, right back side, left inner side, and right inner side of the hand. - A silhouette of the hand facilitates taking photos. - Comments and personal notes can be added to the photos. - The photos are saved locally on the app and assigned a date. Older photos can be selected via a calendar. | Recording and observing the skin condition | At home during the maintenance program | Photos can be taken every day. Once a week, on Sunday, users receive a push notification reminding them to take photos. | The push notification is sent at an individually selected time. |
|  | To listen (German: Hörenswert) | - The episodes describe various strategies that can help with the implementation of skin protection or other difficulties associated with the skin disease. - Four podcast episodes are available for the following topics: (1) episode 1—*Skin protection is all well and good, but how do I manage it in everyday life?*; (2) episode 2—*What actually is stress and what can I do to reduce it?*; (3) episode 3—*What are the internal causes of skin diseases and how can I learn to accept my disease?*; and (4) episode 4—*How can I deal with difficult situations?* | Information about the skin disease, associated difficulties, and strategies | At home during the maintenance program | The first episode is available immediately after registration. The other episodes are activated one after the other every 14 days. | None |
|  | Skin protection 101 (German: Das Hautschutz 1x1) | - Patients can read and see content on the topics of systematic skin protection, itching, and stress. - The content is presented in textboxes and videos in a Q&A^c^ format. | Information about seminar content on skin protection, itching, and stress. | At home during the maintenance program | All content is available right from the start. | None |
|  | My accountabilities (German: Meine Zuständigkeiten) | - It contains information on the tasks of contact persons and institutions involved in the care of insured persons. - The content is presented in a Q&A format. | Information about organizational issues related to care and accountabilities | At home during the maintenance program | All content is available right from the start. | None |
|  | Other functions | - Push notifications that remind of app use. | Reminding users to use the app | At home during the maintenance program | If users have not entered any information into the app for 14 days | None |

^a^SMART: specific, measurable, achievable, realistic, and time bound.

^b^MiA: *Mein Hautschutz im Alltag* (*My skin protection in everyday life*).

^c^Q&A: question and answer.
