# Supplementary material for: User Experience of and Adherence to a Smartphone App to Maintain Behavior Change and Self-Management in Patients With Work-Related Skin Diseases: Multistep, Single-Arm Feasibility Study
Source: JMIR Form Res. 2025 Apr 18;9:e66791. doi: 10.2196/66791 (PMC12048786; doi:10.2196/66791)
Supplement: Multimedia Appendix 2 [file formative_v9i1e66791_app2.docx]

# Multimedia Appendix 2: Results of step 1

## **Table 2.1:** Pros, cons and implications for further development processes resulting from step 1) iterative development and testing of intervention components

| **Group** | **Pros** | **Cons** | **Implications for further development processes** |
| --- | --- | --- | --- |
| **Group 1:**  To listen | - By listening to the podcast episodes, information is absorbed more consciously compared to lectures and written material. - Content is described in detail and technical terms are explained. - Podcasts can be listened at different times according to individual needs. - Volume, listening quality and music at the beginning and end of each episode are perceived positively. - Patients could identify with the content and recognize themselves. | - The voices of the speakers were not immediately distinguishable at first, but you get used to them over time. - The duration (20-25 minutes) is rated by some patients as too long. - The content and recommendations on skin protection are often general and not specialised for each profession. | No changes were made to the podcast episodes. If further podcast episodes are added in the future, the duration should be considered by keeping the episodes shorter. |
| **Group 2:**  Face-to-face goal setting interviews | - The interviews provide new impetus and offer a framework for dealing with new plans for skin protection. - Goals are written down and are therefore not forgotten. - The formulated goals motivate to implement skin protection under optimised conditions after discharge. - Goals created an opportunity to be reminded of the plans again and again in everyday life. - Interviews are individualised according to the patient's needs. - The time frame was appropriate (max. 30 minutes). - The number of goals formulated is manageable (max. 5). - The atmosphere of the interviews and the opportunity to communicate openly and freely are emphasised positively. | - Some patients felt they had been caught taking about mistakes regarding their personal skin protection behaviour. - Some patients wanted to formulate goals in other areas besides skin protection (e.g. dealing with stress). | The category "Others" has been added for individual goals besides skin protection. |
| **Group 3:**  My skin protection behaviour and My accountabilities | *My skin protection behaviour*   - The diagram helped to visualise the own behaviour. - The entry of behaviour was intuitive and can be mostly integrated into everyday life. | *My skin protection behaviour*   - Patients are sometimes under pressure to justify themselves and would like to comment on the recorded behaviour to the clinical staff. - Patients did not always have their smartphones with them, so that the recorded behaviour can only be added in the evening or after work. - Not all patients have received reminders. - The view of the days in the diagram was too small. | *My skin protection behaviour*   - The heading "Overview of my values" was inserted above the diagram to minimise the feeling of justification. - The sending of reminder messages was discussed with and solved by the technicians. - The font size has been increased and the scaling in the diagram has been adjusted. |
|  | *My accountabilities*   - Patients rated the content as trustworthy and would rather research in the app than on the internet. - Information can be looked up quickly. | *My accountabilities*   - Much of the content was already familiar and not new, as patients have been suffering from the skin disease for a longer time. - Patients reported on their experiences finding a specialist dermatologist with experiences in therapy with work-related hand eczema. | *My accountabilities*   - A list of doctors certified in occupational dermatology has been added. |
|  | *General*   - App is simply structured, clear, well illustrative, factually and easy to use. | *General*   - Patients were not familiar with all functions of the app. | *General*   - A video has been created that explains the operation, functions and objectives of the app. |
| **Group 4:**  Skin protection 101 and My skin documentation | *Skin protection 101*   - Information is summarised in a compact form. - Information is easier to understand and access compared to the internet. | *Skin protection 101*  / | *Skin protection 101*  / |
|  | *Skin documentation*   - Taking photos was easy to use and the quality of the pictures is good. - Using the app was simple and intuitive and its structure reminds of other apps. - The comment function in the skin documentation was used for very individual aspects (e.g. documentation of the therapy or the course of the disease). | *Skin documentation*   - Patients would have liked to mark skin areas in the photos. - One patient did not receive any reminder messages. - Photos from the smartphone photo gallery cannot be uploaded to the app. - When entering comments in the photo documentation, every change was always saved so that the text could not be read for a few seconds. | *Skin documentation*   - To test the photo function in the next evaluation steps, the skin areas were initially not marked in order to generate standardised data material. - Patients were informed that access to the gallery is not possible because of data protection reasons. - The comment function has been adapted so that the text is only saved at the end. An additional button for saving has been added. - An informational text was added to the comment function to provide examples and incentives for its use. |
|  | *General*   - The app makes a trustworthy impression. | *General*   - The app is only designed for hand eczema, but not, for example, for foot eczema. | *General*  / |
