# Supplementary material for: User Experience of and Adherence to a Smartphone App to Maintain Behavior Change and Self-Management in Patients With Work-Related Skin Diseases: Multistep, Single-Arm Feasibility Study
Source: JMIR Form Res. 2025 Apr 18;9:e66791. doi: 10.2196/66791 (PMC12048786; doi:10.2196/66791)
Supplement: Multimedia Appendix 3 [file formative_v9i1e66791_app3.docx]

# Multimedia Appendix 3: User experience

**Table 3.1:** Descriptive data for the assessment of the UX with the dimensions trust, content, aesthetic, usability and feelings and emotions

|  | **1**  *lowest rating*  **N (%)** | **2**  **N (%)** | **3**  **N (%)** | **4**  **N (%)** | **5**  *highest rating*  **N (%)** |
| --- | --- | --- | --- | --- | --- |
| **Trust** |  |  |  |  |  |
| Are you concerned that data you have entered into the app could be disclosed to third parties? | - | - | - | 10 (41.7) | 13 (54.2) |
| How high is your trust in the app that your data is securely stored and protected? | - | 1 (4.2) | 5 (21.7) | 12 (52.2) | 5 (21.7) |
| The information in the app appears to come from a credible source. | - | - | - | 10 (43.5) | 13 (56.5) |
|  |  |  |  |  |  |
| **Content** |  |  |  |  |  |
| Are the explanations of graphics or images clear and logical? | - | - | 1 (4.2) | 13 (54.2) | 9 (39.1) |
| The content of the app is comprehensive yet precise. | - | - | 2 (8.7) | 14 (60.9) | 7 (30.4) |
| Are the texts understandable? | - |  | 1 (4.2) | 9 (37.5) | 13 (54.2) |
| Is the content of the app relevant to you? | - | 2 (8.7) | 4 (17.4) | 13 (54.2) | 4 (16.7) |
| **Aesthetic** |  |  |  |  |  |
| How would you rate the overall appearance of the app? | - | - | 3 (13.0) | 14 (60.9) | 6 (26.1) |
| What is the quality and resolution of the images and texts? | - | - | 1 (4.3) | 13 (56.5) | 9 (39.1) |
| **Usability** |  |  |  |  |  |
| Are interactions (tap, swipe, press, scroll) self-explanatory across all views? | - | 1 (4.3) | 4 (17.4) | 12 (52.2) | 6 (26.1) |
| Are interactions (tapping, swiping, pressing, scrolling) consistent across all menu items? | - | - | 2 (8.7) | 10 (43.5) | 11 (47.8) |
| Is the menu navigation logical and does the app have all the necessary links between the pages? | - | 3 (13.0) | 2 (8.7) | 15 (65.2) | 3 (13.0) |
| How clear are the names of the menus or icons? | - | - | 2 (8.7) | 14 (60.9) | 7 (30.4) |
| How easy was it for you to learn how to use the app? | 1 (4.2) | - | 4 (16.7) | 9 (37.5) | 10 (41.7) |
| How well and quickly do the app's applications (buttons, menus) respond? | - | - | 1 (4.2) | 12 (50.0) | 11 (45.8) |
| **Feelings and emotions** |  |  |  |  |  |
| Does the app include interactive features (e.g. user input, feedback, automatic prompts)? | - | - | 2 (8.7) | 12 (52.2) | 9 (39.1) |
| The app offers me sufficient options to customise it to my personal requirements and needs. | - | 1 (4.2) | 8 (33.3) | 12 (50.0) | 3 (12.5) |
| Is the information presented in an interesting way? | - | - | 6 (25.0) | 16 (66.7) | 2 (8.3) |
| The app contains functions that make it entertaining. | - | 2 (8.3) | 13 (54.2) | 9 (37.5) | - |
| I find the app entertaining. | - | - | 15 (62.5) | 9 (37.5) | - |

**Table 3.2:** Descriptive data from the scores of the UX and UX-dimensions

| **Dimension**  **(number of items)** | **Theoretical**  **Min. / Max.** | **Actual**  **Min. / Max.** | **M (SD)** | **Md** | **IQR** |
| --- | --- | --- | --- | --- | --- |
| Feelings and emotions (5) | 5 / 25 | 15 / 22 | 17.57 (2.09) | 17.00 | 3.0 |
| Content (4) | 4 / 20 | 11 / 20 | 16.91 (2.09) | 17.00 | 2.0 |
| Usability (6) | 6 / 30 | 15 / 30 | 25.00 (3.53) | 26.00 | 3.0 |
| Aesthetic (2) | 2 / 10 | 6 / 10 | 8.48 (1.04) | 8.00 | 1.0 |
| Trust (3) | 3 / 15 | 8 / 15 | 12.22 (1.83) | 12.00 | 2.0 |
| User Experience (20) | 20 / 100 | 57 / 95 | 80.18 (8.94) | 80.00 | 11.8 |

M=mean; Md=median; SD=standard deviation; Min=minimum; Max.=maximum; IQR=interquartile range
