# Supplementary material for: User Experience of and Adherence to a Smartphone App to Maintain Behavior Change and Self-Management in Patients With Work-Related Skin Diseases: Multistep, Single-Arm Feasibility Study
Source: JMIR Form Res. 2025 Apr 18;9:e66791. doi: 10.2196/66791 (PMC12048786; doi:10.2196/66791)
Supplement: Multimedia Appendix 4 [file formative_v9i1e66791_app4.docx]

# Supplement Material 4: Subjective quality and perceived impact

**Table 4.1:** Results of the question “Would you recommend the app to other people with work-related skin diseases, e.g. colleagues?”

|  | **Frequency (N)** | **Percent (%)** | **Valid**  **Percent (%)** | **Cumulative percent (%)** |
| --- | --- | --- | --- | --- |
| Definitely not | - | - | - | - |
| Rather not | - | - | - | - |
| Maybe | 6 | 25.0 | 25.0 | 25.0 |
| Rather yes | 10 | 41.7 | 41.7 | 66.7 |
| Definitely yes | 8 | 33.3 | 33.3 | 100.0 |
| Total | 24 | 100.0 | 100.0 |  |

**Table 4.2:** Results of the question “How often would you use the app in the next 12 months?”

|  |  | **Frequency (N)** | **Percent (%)** | **Valid**  **Percent (%)** | **Cumulative percent (%)** |
| --- | --- | --- | --- | --- | --- |
|  | Never | 4 | 16.7 | 17.4 | 17.4 |
|  | Approx. once a month | 3 | 12.5 | 13.0 | 30.4 |
|  | Approx. every fortnight | 1 | 4.2 | 4.3 | 34.8 |
|  | Approx. once a week | 7 | 29.2 | 30.4 | 65.2 |
|  | Several times a week | 6 | 25.0 | 26.1 | 91.3 |
|  | Once a day | 1 | 4.2 | 4.3 | 95.7 |
|  | Several times a day | 1 | 4.2 | 4.3 | 100.0 |
|  | Total | 23 | 95.8 | 100.0 |  |
| Missing | -999 | 1 | 4.2 |  |  |
| Total |  | 24 | 100.0 |  |  |

**Table 4.3:** Results of the question “Would you pay for this app?”

|  | **Frequency (N)** | **Percent (%)** | **Valid**  **Percent (%)** | **Cumulative percent (%)** |
| --- | --- | --- | --- | --- |
| Definitely not | 4 | 16.7 | 16.7 | 16.7 |
| Rather not | 10 | 41.7 | 41.7 | 58.3 |
| Maybe | 10 | 41.7 | 41.7 | 100.0 |
| Rather yes | - | - | - | - |
| Definitely yes | - | - | - | - |
| Total | 24 | 100.0 | 100.0 |  |

**Table 4.4:** Results of the question “How many stars would you rate the app with?”

|  |  | **Frequency (N)** | **Percent (%)** | **Valid**  **Percent (%)** | **Cumulative percent (%)** |
| --- | --- | --- | --- | --- | --- |
|  | * | - | - | - | - |
|  | ** | - | - | - | - |
|  | *** | 4 | 16.7 | 17.4 | 17.4 |
|  | **** | 15 | 62.5 | 65.2 | 82.6 |
|  | ***** | 4 | 16.7 | 17.4 | 100.0 |
|  | Total | 23 | 95.8 | 100.0 |  |
| Missing | -999 | 1 | 4.2 |  |  |
| Total |  | 24 | 100.0 |  |  |

**Table 4.5:** Results of the items regarding the perceived impact of the MiA-app

|  | **Do not agree at all** | **Rather disagree** | **In some cases** | **Aagree somewhat** | **Agree completely** |
| --- | --- | --- | --- | --- | --- |
|  | N (%) | N (%) | N (%) | N (%) | N (%) |
| The app makes me aware of how important it is to occupy at my own skin protection behaviour | - | - | 1 (4.2) | 9 (37.5) | 12 (50.0) |
| The app increases my knowledge of skin protection behaviour | - | 1 (4.2) | 2 (8.3) | 11 (45.8) | 8 (33.3) |
| The app motivates me to improve my skin protection behaviour | - | 2 (8.3) | 4 (16.7) | 9 (37.5) | 7 (29.2) |
| The app encourages me to seek further support and make use of help on skin protection behaviour if I need help | - | 2 (8.3) | 7 (29.2) | 8 (33.3) | 5 (20.8) |
| The app enables me to implement my skin protection behaviour even better | - | - | 5 (20.8) | 11 (45.8) | 6 (25.0) |
