# Supplementary material for: User Experience of and Adherence to a Smartphone App to Maintain Behavior Change and Self-Management in Patients With Work-Related Skin Diseases: Multistep, Single-Arm Feasibility Study
Source: JMIR Form Res. 2025 Apr 18;9:e66791. doi: 10.2196/66791 (PMC12048786; doi:10.2196/66791)
Supplement: Multimedia Appendix 5 [file formative_v9i1e66791_app5.docx]

# Multimedia Appendix 5: Adherence to the app’s functions *My skin protection behaviour*, *My skin documentation* and *My skin protection goals*

**Table 5.1:** Total of adherent and non-adherent people in the app function *My skin protection behaviour*

|  | **hand cleansing** | | **skin protection** | | **skin care** | |
| --- | --- | --- | --- | --- | --- | --- |
| **day** | *Adherent*  *(n)* | *Nonadherent (n)* | *Adherent*  *(n)* | *Nonadherent (n)* | *Adherent*  *(n)* | *Nonadherent*  *(n)* |
| 1 | 28 | 14 | 13 | 29 | 28 | 14 |
| 2 | 34 | 8 | 18 | 24 | 33 | 9 |
| 3 | 32 | 10 | 16 | 26 | 32 | 10 |
| 4 | 27 | 15 | 18 | 24 | 27 | 15 |
| 5 | 25 | 17 | 14 | 28 | 26 | 16 |
| 6 | 28 | 14 | 13 | 29 | 28 | 14 |
| 7 | 28 | 14 | 17 | 25 | 29 | 13 |
| 8 | 23 | 19 | 13 | 29 | 23 | 19 |
| 9 | 25 | 17 | 16 | 26 | 26 | 16 |
| 10 | 24 | 18 | 14 | 28 | 24 | 18 |
| 11 | 25 | 17 | 14 | 28 | 24 | 18 |
| 12 | 22 | 20 | 12 | 30 | 22 | 20 |
| 13 | 23 | 19 | 11 | 31 | 23 | 19 |
| 14 | 23 | 19 | 15 | 27 | 23 | 19 |
| 15 | 23 | 19 | 16 | 26 | 23 | 19 |
| 16 | 25 | 17 | 12 | 30 | 25 | 17 |
| 17 | 22 | 20 | 12 | 30 | 22 | 20 |
| 18 | 24 | 18 | 13 | 29 | 24 | 18 |
| 19 | 23 | 19 | 10 | 32 | 22 | 20 |
| 20 | 24 | 18 | 10 | 32 | 23 | 19 |
| 21 | 21 | 21 | 9 | 33 | 21 | 21 |
| 22 | 23 | 19 | 15 | 27 | 23 | 19 |
| 23 | 24 | 18 | 18 | 24 | 24 | 18 |
| 24 | 23 | 19 | 19 | 23 | 23 | 19 |
| 25 | 21 | 21 | 17 | 25 | 20 | 22 |
| 26 | 18 | 24 | 14 | 28 | 18 | 24 |
| 27 | 22 | 20 | 12 | 30 | 22 | 20 |
| 28 | 22 | 20 | 16 | 26 | 23 | 19 |
| 29 | 19 | 23 | 13 | 29 | 19 | 23 |
| 30 | 18 | 24 | 15 | 27 | 19 | 23 |
| 31 | 21 | 21 | 16 | 26 | 20 | 22 |
| 32 | 19 | 23 | 15 | 27 | 19 | 23 |
| 33 | 20 | 22 | 12 | 30 | 20 | 22 |
| 34 | 20 | 22 | 11 | 31 | 19 | 23 |
| 35 | 18 | 24 | 13 | 29 | 18 | 24 |
| 36 | 20 | 22 | 16 | 26 | 20 | 22 |
| 37 | 19 | 23 | 16 | 26 | 19 | 23 |
| 38 | 19 | 23 | 15 | 27 | 18 | 24 |
| 39 | 20 | 22 | 16 | 26 | 20 | 22 |
| 40 | 19 | 23 | 12 | 30 | 18 | 24 |
| 41 | 20 | 22 | 10 | 32 | 18 | 24 |
| 42 | 17 | 25 | 14 | 28 | 17 | 25 |
| 43 | 19 | 23 | 15 | 27 | 19 | 23 |
| 44 | 18 | 24 | 14 | 28 | 18 | 24 |
| 45 | 17 | 25 | 15 | 27 | 17 | 25 |
| 46 | 20 | 22 | 15 | 27 | 20 | 22 |
| 47 | 15 | 27 | 10 | 32 | 14 | 28 |
| 48 | 17 | 25 | 9 | 33 | 17 | 25 |
| 49 | 17 | 25 | 14 | 28 | 17 | 25 |
| 50 | 20 | 22 | 18 | 24 | 19 | 23 |
| 51 | 17 | 25 | 17 | 25 | 16 | 26 |
| 52 | 17 | 25 | 16 | 26 | 17 | 25 |
| 53 | 17 | 25 | 14 | 28 | 17 | 25 |
| 54 | 15 | 27 | 10 | 32 | 15 | 27 |
| 55 | 18 | 24 | 9 | 33 | 17 | 25 |
| 56 | 16 | 26 | 12 | 30 | 16 | 26 |
| 57 | 19 | 23 | 17 | 25 | 19 | 23 |
| 58 | 16 | 26 | 14 | 28 | 16 | 26 |
| 59 | 16 | 26 | 15 | 27 | 15 | 27 |
| 60 | 17 | 25 | 14 | 28 | 17 | 25 |
| 61 | 17 | 25 | 14 | 28 | 16 | 26 |
| 62 | 17 | 25 | 11 | 31 | 17 | 25 |
| 63 | 15 | 27 | 12 | 30 | 15 | 27 |
| 64 | 15 | 27 | 13 | 29 | 15 | 27 |
| 65 | 15 | 27 | 13 | 29 | 15 | 27 |
| 66 | 17 | 25 | 16 | 26 | 17 | 25 |
| 67 | 17 | 25 | 15 | 27 | 15 | 27 |
| 68 | 15 | 27 | 12 | 30 | 15 | 27 |
| 69 | 15 | 27 | 10 | 32 | 15 | 27 |
| 70 | 16 | 26 | 12 | 30 | 16 | 26 |
| 71 | 15 | 27 | 14 | 28 | 15 | 27 |
| 72 | 14 | 28 | 13 | 29 | 14 | 28 |
| 73 | 16 | 26 | 14 | 28 | 16 | 26 |
| 74 | 16 | 26 | 15 | 27 | 16 | 26 |
| 75 | 15 | 27 | 9 | 33 | 15 | 27 |
| 76 | 15 | 27 | 10 | 32 | 15 | 27 |
| 77 | 16 | 26 | 13 | 29 | 16 | 26 |
| 78 | 16 | 26 | 15 | 27 | 16 | 26 |
| 79 | 14 | 28 | 11 | 31 | 14 | 28 |
| 80 | 16 | 26 | 14 | 28 | 16 | 26 |
| 81 | 16 | 26 | 15 | 27 | 16 | 26 |
| 82 | 14 | 28 | 9 | 33 | 14 | 28 |
| 83 | 16 | 26 | 9 | 33 | 16 | 26 |
| 84 | 12 | 30 | 9 | 33 | 12 | 30 |
| Mean | 19.43 | 22.57 | 13.54 | 28.46 | 19.26 | 22.74 |

**Table 5.2:** Total of adherent and non-adherent people in the app function *My skin documentation* and *My skin protection goals*

|  | **My skin documentation** | | **My skin protection goals** | |
| --- | --- | --- | --- | --- |
| **week** | *Adherent* (n) | *Nonadherent* (n) | *Adherent* (n) | *Nonadherent* (n) |
| 1 | 10 | 32 | 4 | 38 |
| 2 | 9 | 33 | 2 | 40 |
| 3 | 8 | 34 | 4 | 38 |
| 4 | 10 | 32 | 3 | 39 |
| 5 | 9 | 33 | 5 | 37 |
| 6 | 6 | 36 | 4 | 38 |
| 7 | 4 | 38 | 3 | 39 |
| 8 | 6 | 36 | 6 | 36 |
| 9 | 6 | 36 | 5 | 37 |
| 10 | 4 | 38 | 5 | 37 |
| 11 | 2 | 40 | 0 | 42 |
| 12 | 4 | 38 | 4 | 38 |
| Mean | 6.5 | 35.5 | 3.75 | 38.25 |
